# The three paradoxes of patient flow: an explanatory case study

**DOI:** 10.1186/s12913-017-2416-8

**Published:** 2017-07-12

**Authors:** Sara A. Kreindler

**Affiliations:** Department of Community Health Sciences and Health Systems Performance Platform, George & Fay Yee Centre for Healthcare Innovation, University of Manitoba, 451-753 McDermot Ave, Winnipeg, MB R3E 0T6 Canada

**Keywords:** Patient flow, Health services organization and administration, Organizational efficiency, Qualitative research

## Abstract

**Background:**

Health systems in many jurisdictions struggle to reduce Emergency Department congestion and improve patient flow across the continuum of care. Flow is often described as a systemic issue requiring a “system approach”; however, the implications of this idea remain poorly understood. Focusing on a Canadian regional health system whose flow problems have been particularly intractable, this study sought to determine what system-level flaws impede healthcare organizations from improving flow.

**Methods:**

This study drew primarily on qualitative data from in-depth interviews with 62 senior, middle and departmental managers representing the Region, its programs and sites; quantitative analysis of key flow indicators (1999–2012) and review of ~700 documents furnished important context. Examination of the interview data revealed that the most striking feature of the dataset was contradiction; accordingly, a technique of dialectical analysis was developed to examine observed contradictions at successively deeper levels.

**Results:**

Analysis uncovered three paradoxes: “Many Small Successes and One Big Failure” (initiatives improve parts of the system but fail to fix underlying system constraints); “Your Innovation Is My Aggravation” (local innovation clashes with regional integration); and most critically, “Your Order Is My Chaos” (rules that improve service organization for my patients create obstacles for yours). This last emerges when some entities (sites/hospitals) define their patients in terms of their location in the system, while others (regional programs) define them in terms of their needs/characteristics. As accountability for improving flow was distributed among groups that thus variously defined their patients, local efforts achieved little for the overall system, and often clashed with each other. These paradoxes are indicative of a fundamental antagonism between the system’s parts and the whole.

**Conclusion:**

An accretion of flow initiatives in all parts of the system will never add up to a system approach, and may indeed perpetuate the paradoxes. What is needed is a coherent strategy of defining patient populations by needs, analyzing their entire trajectories of care, and developing consistent processes to better meet those needs.

## Background

Patient flow – ensuring that patients receive the care they need, when and where they need it – is one of the greatest challenges facing healthcare today [1–3]. Stagnant flow has myriad destructive consequences: for patients, delayed care, with protracted suffering, anxiety and risk; for providers, stress, overload, and burnout; for the health system, reduced quality and sustainability. While Emergency Department (ED) congestion is its most obvious symptom, poor flow may reflect problems at other points along the continuum of care, such as a lack of available inpatient beds (which may in turn reflect a lack of available long-term care spaces), or weak primary care infrastructure. Widespread recognition of this fact has prompted numerous calls for a “system approach”; unfortunately, this term is often used rather superficially, to denote merely that flow improvement requires intervention in more than one part of the system. A genuine systems approach begins with analysis of how system *design* generates the observed patterns of interaction among system parts; [4] only then is it possible to determine which parts, in which sequence, should be the target of what intervention.

This paper is informed by the concept of complex adaptive systems (CAS) – systems whose many moving parts interact in unpredictable ways, producing patterns that emerge only at the level of the whole [5]. A growing body of literature characterizes healthcare systems in this way; this literature reflects two competing paradigms, each of which gives rise to a different approach to system improvement. The first focuses on the fact that multifarious system behavior can be traced back to a few rules/parameters; improvement is seen as a matter of precisely identifying the problematic parameter and implementing a carefully designed response [4, 6]. The second focuses on the equally important fact that system behavior is non-linear and often erratic, making it difficult to intervene with precision; from this perspective, improvers must encourage a diversity of potentially useful responses, providing “simple guiding principles” rather than detailed specifications [7, 8]. This paper proceeds from the premise that both paradigms are valuable and must be held in dynamic tension.

Using a complex-systems lens, this explanatory case study [9] sought to understand the causes of a regional health system’s intractable difficulty in improving patient flow. The study considered the issue at both the micro (initiative-by-initiative) and macro (whole-system) levels; this article concerns the latter (a companion article reports the former), [10] with the specific research question: What are the system-level flaws or contradictions that thwart efforts to improve flow? It will examine the issue at successively deeper levels, ultimately exposing a fundamental antagonism between the parts of the system and the whole.

Sound principles for flow improvement – and indeed, the “patient flow” metaphor itself – have been imported from the discipline of operations management. Operations research has identified two deductive laws: the law of variability, which derives from queuing theory, and the law of bottlenecks, which is articulated by the theory of constraints [11]. The law of variability states that the amount of variability present in a process (be it from internal or external sources) is inversely related to the process’ efficiency. This is because the more variability is present, the higher the peaks in utilization, thus the larger the buffers – inventory, spare capacity, and/or waiting time – required to manage them [12]. The law of bottlenecks states that a process’ overall efficiency can be improved only by addressing its major bottleneck or “constraint”; [13] if improvement occurs at non-constraint steps, people or materials will advance more quickly only to pile up at the bottleneck as badly as before. The two laws offer complementary insights, making it possible to identify both how and where to intervene for maximal improvement. To account for them, Schmenner and Swink (1998) proposed the Theory of Swift, Even Flow: the more swift and even the flow of materials through a process, the more productive (efficient) that process is [11].

These principles, and improvement methodologies derived from them, are widely known and used in healthcare. In practice, however, the benefits remain elusive. The evidence base on specific flow initiatives is disappointing: even among those few interventions that have been confirmed as effective, impacts are highly variable and seldom dramatic (see de Grood et al.’s 2012 systematic review; [14] see also the overview of reviews in the companion article) [10]. Lean, the most widely used process-improvement methodology, appears to achieve significant, sustained gains only when applied to relatively simple processes, [15] suggesting either that many healthcare processes are too technically and socially complex to be amenable to variability-curtailment efforts, [7] that Lean has not been used to its potential (i.e., to detect and eliminate not merely obvious waste but subtle sources of variability), [12] or both. Moreover, it has been observed that whereas the manufacturing sector’s use of Lean has evolved from the small-scale application of “tools” to the system-wide application of principles, such evolution has not yet occurred in healthcare [16]. It is essential to understand what impedes healthcare systems from successfully undertaking the system-level analysis and action required to achieve large-scale improvement [10–13, 17]. Accordingly, this article reflects a shift in focus from the level of flow initiatives to that of system design and administration.

## Method

### Context

In Canada, healthcare is a provincial responsibility, and most provinces have devolved its planning and administration to regional health authorities. Care is publicly *financed*, but *delivered* by a patchwork of organizations and individuals with varying degrees of connection to the system. The regional health system studied (“the Region”) serves a large western city and its surrounding area, and has a matrix structure in which programs (e.g., Medicine, Surgery, Emergency, Home Care) cut across sites (hospitals and community areas). Half of its six hospitals have dissolved their boards and become operating divisions of the Region; in practice, all are expected to be accountable to the Region but have global budgets and considerable autonomy in their activities. Most ambulatory care is provided by independent, fee-for-service physicians, and most nursing homes are privately owned (non- or for-profit).

Compared to its western urban peers, the Region performs exceptionally poorly on metrics of both ED and inpatient flow. Patient flow has been the object of intense focus for more than a decade, with myriad improvement efforts at the site, program and regional levels. Certain of these initiatives have demonstrated local impacts, yet systemwide, there has been no appreciable decline in either ED or inpatient length of stay (LOS). Recognizing that patient flow was emerging as a major regional priority, the Region’s “embedded” research unit (which specializes in collaborative, problem-driven research) offered to undertake an investigation of why past efforts to improve flow had fallen so far short of expectations. The analysis paid special attention to EDs, where the symptoms to be explained were most acute, but sought causes at the level of overall system design.

### Methodology

The research question was ideally suited to case study methodology, which entails the “investigation of a phenomenon within its real-life context.” [9] The *explanatory* case study approach, which is concerned with the identification of underlying causal mechanisms, reflected a critical realist epistemology [18]. The study’s quantitative component, which confirmed the lack of regional flow improvement and the negligible impact of most interventions, is reported as a supplement to the companion article [10]. This paper focuses on the study’s qualitative component; in-depth individual and small-group interviews (early 2013) with a purposive sample of 62 senior, middle and departmental managers constituted the primary method, supplemented by review of all available documents describing the nature, implementation, and/or impacts of flow initiatives.

Interviews focused on managers, who are best situated to observe system-level phenomena, as they interact with other parts of the system to an extent that frontline staff typically do not; they are also party to regional-level decision-making processes. Participants were recruited on the basis of current or past organizational role and through snowball sampling; recruitment concluded once the sample reflected all key groups (the Region, all 6 hospitals, several community areas, and the programs with the greatest involvement in flow efforts) and the data were reaching saturation. Only one person (a former employee) declined an interview; four did not reply to the e-mail. Over two thirds of participants had a clinical background, most commonly nursing; one fifth were physicians, most of whom maintained a clinical practice.

Table 1 presents the semi-structured guide for the one-hour interviews; in practice, most participants needed minimal prompting to discuss flow issues in great depth (some spoke for up to 20 min uninterrupted in response to the introductory question). Accordingly, I typically let the participant lead the conversation, creating a more unstructured format in which my main contribution was to request elaboration and clarification. All but one participant agreed to be audiotaped.

**Table 1:** Interview Guide

1. Could you start by telling me about your role and your involvement in improving flow?
• Probe: How long have you been in this role? (Ask about past roles in the organization if applicable.)
2. Please describe [XYZ project, as applicable] and your role in it.
• How did you choose this particular initiative? (What problem were you trying to solve? Where did you get the idea? Who was involved in the decision?)
• Can you walk me through the process of implementing the project?
• What worked well? What didn’t work well?
• Did you encounter major barriers to implementing the project? If so, what were they? What caused them? How did you address them?
• What were the project’s outcomes? Why do you think this occurred?
• Have you tried to spread the project beyond [area]? How has that gone?
3. Now looking at the regional level: overall, how do you feel efforts to improve patient flow are going?
• Probes: What has worked well? What hasn’t? Why? (Ask for examples.)
• What do you think are the most important things the Region should do to improve flow? (What would that look like? What would it take?)
• How important do you think it is for all the hospitals to have similar processes or similar initiatives for improving flow?
• What do you think should be the role of (programs, sites) in improving flow? Why?
4. Is there anything else we should know?
5. Is there anyone else we should talk to?

Analysis relied on the constant comparative method [19]. During the interview process, I made extensive notes and began to identify potential themes; afterwards, I began open-coding the verbatim transcripts, seeking to capture all expressed opinions and recommendations, and summarizing these on sticky notes color-coded by affiliation (hospital, Emergency, inpatient program, community, regional). I experimented with various ways of organizing the codes into themes, and settled on a working coding scheme about halfway through the transcripts. I then extracted relevant quotations into a Microsoft Excel spreadsheet to allow sorting and re-sorting. The analytic process continued to be iterative, moving back and forth between extracted quotations, full transcripts, documentary and quantitative data sources (to verify facts and test interpretations), relevant literature, and the evolving coding scheme.

In the course of analysis, I discovered that the most salient feature of the dataset was paradox: Stakeholders passionately defended accounts that were not merely divergent, but incompatible; what one person embraced as a solution would, according to another, be disastrous. Upon reflection on the content and mode of expression (e.g., animated tone, emotive language, jokes about getting in trouble for one’s comments), I came to believe that each participant was expressing what s/he could “see” from his/her vantage point, and that triangulation (with all the original, navigational implications of the term) would be key to revealing the nature of the landscape. The crucial question, then, became: What structural reality could give rise to such profound contradictions within the same system, sometimes within the same building? However, this question did not lend itself easily to standard methods of qualitative analysis. Discourse analysis, while often suitable for exploring contradictions, is concerned with the performative aspect of speech, eschewing the assumption that its content reveals something about the speaker’s internal (let alone external) world [20, 21]. Interpretative phenomenological analysis is integrally concerned with the participant’s internal world, but regards the understanding thereof as an end in itself; this idiographic approach would preclude combining divergent interpretations in order to draw inferences about their object [22]. Less interpretive methods typically stop short at describing the contrasting viewpoints, or explain the conflicts in terms of manifest social categories (e.g., physicians *vs.* nurses, managers *vs.* staff), [23] whereas in this case the observed pattern of disagreements had no obvious explanation. Accordingly, I developed a *dialectical technique*; this involves identifying the contradictory propositions (*thesis* and *antithesis*) that make up each paradox, and examining them in detail in order to derive a *synthesis* that can encompass both. Within each thematic category (defined by a central conflict or paradox), the following guiding questions were used iteratively to interrogate the data:

1. What are the *thesis* and *antithesis*?

- In what examples are they revealed?
- Who advocates positions on each side of the dialectic? If there are intergroup divisions, how might each group’s position in the system have shaped their perspectives?
- Are there any points of convergence or anomalies, such as internal contradictions in what some participants advocate?
  2. What do these patterns reveal about the axis of conflict?
  3. What *synthesis* could encompass both thesis and antithesis? Is it a principle that could be the basis for consensus, or does it express a structural contradiction (i.e., a reason why conflict is built into the system), pointing to a need for system change?

The articulation and scrutiny of participants’ competing perspectives is in keeping with the constant comparative method, and did not require any unusual practices during the coding process. What the dialectical technique added was the use of logical inference to identify an axis of conflict and a synthesis.

The literature affirms contradiction to be a normal and benign feature of organizational life, reflecting the dynamic tension between such opposing elements as cooperation and competition or care and cure [7, 24]. Within the dataset, some paradoxes were indeed of this nature (e.g., efficiency *vs.* patient-centeredness; capacity *vs*. efficiency). Others, however, appeared to be of a different order: rather than manifesting the perennial ebb and flow between polarities, these paradoxes exposed a structural antagonism between the parts of the system and the whole. It is this latter group with which this article concerns itself.

The findings discussed in this and related articles were first released to the Region in an internal report. A draft was shared with all participants, several of whom remarked that it captured the major issues and the range of stakeholder perspectives; some commented on the interpretations or raised additional issues, and a few made factual corrections. Feedback was carefully considered and incorporated into the final version.

The focus on paradoxes and the dialectical technique created an analytic experience akin to peeling an onion; some contradictions did not become apparent until others had been interpreted. For this reason, results and discussion cannot be entirely segregated; rather, exposition and interpretation must be presented in alternating fashion. To ensure the transparency of the analysis, interpretive passages will be marked with the subhead “Towards a Synthesis” (preliminary effort to formulate a synthesis, to be tested against the data) or “Discussion” (final synthesis and its implications).

## Results and discussion

The analysis uncovered three paradoxes, each revealing a deeper stratum of system dysfunction. Table 2 presents a description of each thesis–antithesis pair, the codes from which it emerged, and the considerations used to derive a synthesis.

**Table 2:** Derivation of the Three Paradoxes

Paradox 1 (Many Small Successes and One Big Failure)		
	Thesis	Antithesis
Codes	Many valuable initiatives	Initiatives have low overall impact
	Incremental progress	Lack of progress
	Success stories	“Band-Aid solutions”
	Focus on sphere of control	Problems are outside our control
	No bad initiatives (“everything works”)	Inadequate analysis of problem
		Need for system redesign
Theme	Localized initiatives (= successes)	Localized initiatives (= failure)
Advocated by	Leaders of localized initiatives	Emergency stakeholders
	Sites active in flow efforts	Sites less active in flow efforts
	Regional managers with major responsibility for current flow effort	Some program leaders of flow efforts
		Regional managers without major responsibility for current flow effort
Points of Convergence, Anomalies	Proponents of the antithesis themselves drew attention to the conjunction of localized improvements and stagnant system performance. Both sides noted the difficulty of working as a system, describing power struggles, unclear accountabilities and lack of integration.	
Axis of Conflict	Focus on system parts *vs*. whole.	
Synthesis	Initiatives have improved parts of the system but missed the greatest system problems/constraints.	
Paradox 2 (Your Innovation Is My Aggravation)		
	Thesis	Antithesis
Codes	Region stifles innovation	Site “innovations” undermine or duplicate program strategies
	Regional/program change processes are slow, cumbersome	
		Sites’ efforts are hasty, unsystematic
	Sites should be allowed to find different ways to destination	
		Site initiatives contradict each other (different destinations)
	Pan-regional consistency less important than flexibility	
		Pan-regional consistency essential for efficiency, equity
	Region/program wants to control	
		Sites want to be unique/special
Theme	Site-led innovation (desirable)	Site-led innovation (undesirable)
Advocated by	Site stakeholders	Leaders of most programs
		Most regional managers
Points of Convergence, Anomalies	Participants on both sides advocated the spread of best practices through tailoring to local context; however, any examples provided were typically not flow-related. When participants described desirable/acceptable flow-related practice, sites’ definitions were broader than programs’.	
Axis of Conflict	Decentralization *vs*. centralization	
Synthesis	If sites and regional programs shared clear, specific goals (not merely general aspirations), either could lead change.	
Paradox 3 (Your Order Is My Chaos)		
	Thesis	Antithesis
Codes	Somebody else’s rules are the problem (inpatient, community programs; nursing homes, etc.)	Our rules are essential for safety and efficiency (inpatient, community programs)
	Programs’ criteria too restrictive, lead to stateless patients	Programs know whom they can and should serve
	“Off-servicing” is necessary	Off-servicing is detrimental
	Caring for all patients, irrespective of characteristics	Designing services for a defined population
	Service consolidation across sites harms patients	Service consolidation across sites benefits patients
Theme	Gates (should be weakened)	Gates (must be maintained)
Advocated by	Site stakeholders	Leaders of most other programs
	Emergency stakeholders	
Points of Convergence, Anomalies	Participants on both sides recognized that “gates” facilitate programs’ organization of care.	
	Several site and Emergency stakeholders advocated the thesis in relation to other parts of the system, and the antithesis in relation to their own. In contrast, non-Emergency program stakeholders who argued for the antithesis did so consistently.	
Axis of Conflict	Defining patients by location *vs.* by characteristics/needs	
Synthesis	The phenomenon of stateless patients reflects haphazard system design. A well-designed system features appropriate (gated) services to meet the needs of *each* patient population.	

### Paradox #1: Many Small Successes and One Big Failure

#### Thesis and antithesis

From participants’ accounts of past flow efforts, a curious antinomy emerged: the Region’s record appeared to simultaneously instantiate both many small successes and one big failure. Almost every initiative with some evidence base – minor treatment areas, triage nurse ordering, observation units, care maps, discharge facilitators, discharge planning (and numerous enhancements to improve its quality), overcapacity protocols, and many more – had been introduced, or at least attempted, somewhere in the Region; many of these had shown localized positive impacts. When asked about the success of various interventions, most participants gave the impression that everything had worked; few were able to identify any initiatives that had been discontinued due to low effectiveness (“I don’t know if there’s anything that’s completely, we’ve just said, ‘this does not work’ and we’ve turned it away; most of the things we’ve integrated”). In contrast, when asked about the success of flow-improvement efforts overall, participants frequently gave the impression that *nothing* had worked (“I won’t say [regional flow efforts] haven’t gone anywhere but I don’t think they’ve gone very far”).

#### Towards a synthesis

The only way both representations can be accurate is if past initiatives succeeded at improving some part of the system, but failed to fix the underlying system problem(s) or constraint(s). This idea was in fact articulated by several participants:

- “To be honest, nothing’s worked all that well in a global sense. Well, there’ve been a number of...measurably successful initiatives, but if you go into the emergency department, today, as opposed to ten years ago, you wouldn’t really see any appreciable difference.”
- “[There] might have been internal successes, but...we haven’t improved the constraint, we haven’t improved the system.”

If the accretion of changes to discrete parts of the system has, in ten years, brought the Region no closer to solving its system problems, does this imply a need for whole-system redesign? For a handful of participants, this is exactly what it implied:

- “You have to be open to massive model redesign. I don’t believe in the kind of half-assed quality improvement crap that goes on in the hospitals.”
- “You gotta kind of destroy it to figure out how to rebuild it... I just keep hearing resonating in my head, ‘tear down that wall, Mr. Gorbachev!’”

On the other hand, not all participants who criticized the disconnected nature of past efforts went on to advocate redesign. For instance, one senior leader suggested that “an integrated approach to the flow question” would consist of holding each program accountable for improving its own efficiency:

- “So the question then becomes what’s the responsibility…of Surgery, what’s the responsibility of Emergency, what’s the responsibility of Home Care. …If [Home Care is] responsible for pulling patients from the hospital…what would be a good target [for the time required to establish a service plan]? Okay, that’s our target…now let’s talk about what you need to do to achieve that.”

Another participant believed the key lay in instituting shared processes, whereby “all hospitals and services use a similar framework to move patients through the system. So that instead of patients moving through like a Slinky, ‘hurry up and wait’…the analogy is more… hanging onto the rope together and pulling the patient through in a more integrated way.”

However, the greatest proportion of those who invoked systems discourse did so to blame someone else for the flow problem:

- “Part of the reason why [our hospital’s efforts have] been mostly I would say unsuccessful is that we don’t have any authority or control over areas that go beyond the hospital. And the flow issues are systemic, they’re not just belonging to one sector.”
- “My frustration is that everybody keeps looking at Emerg to fix it...we need to look at root cause, and Emergency is not the root cause.”

Such narratives, predictably, elicited frustration from the Region:

- “I was just astounded at some of the comments coming out of people, like at a [leadership] level...they were just saying stuff that, to me, sounds like excuses... And so what I was trying to do was challenge back about, ‘Okay, what’s in your locus of control, though, that you could influence and change right now?’ ...They were pretty quiet.”

#### Discussion

The apparent consensus that flow was a system problem belied great dissension as to what might constitute a system solution. In the absence of a clear, shared vision for system change, discussion of system issues easily degenerated into finger-pointing; impatience with this unproductive dynamic provoked the Region to direct programs and sites to pull back to their respective spheres of control, effectively shutting down system-level exploration. Yet such an approach virtually guarantees that improvement efforts will consist of trivial modifications to non-constraint steps – exactly the approach that has left the Region with Many Small Successes and One Big Failure.

To ascertain what a true system solution might entail, it is necessary to look more closely at issues of accountability and organizational structure. First, it might be observed that systemic change demands a coherent, systemwide response, which in turn demands clear roles and accountabilities. Within the region’s matrix structure – with its six semi-autonomous sites intersected by twenty regional programs – such clarity did not prevail. The Region’s official Accountability Framework holds programs responsible for strategic planning, standards and quality; sites for operations – in practice, however, these domains frequently intersect. Many participants reported endemic role confusion (“the matrix is very confusing to a lot of people”; “there’s no real sense about who’s actually responsible for making [a] decision…who’s accountable”). Beyond this, they described an ongoing site–program power struggle, in which both clung to the broadest possible interpretation of their roles (“some of it is purposeful, not wanting to understand – it’s about control, power”). This brings us to the second paradox.

### Paradox #2: Your Innovation Is My Aggravation

#### Thesis and antithesis

A major area of site–program conflict concerned the tension between local innovation and regional integration. The most common framing of this issue was in terms of the spread of best practice, which some participants thought had been impeded by an overemphasis on site uniqueness:

- “We still continue to waste resources by doing special things at each site, because it’s got to fit their culture – which is really unfortunate, because it just means rework.”

Others countered that the spread of best practice demands an allowance for site uniqueness:

- “Adoption isn’t because of spread, spread is because of the adoption process. It’s different in every site...”
- “We all see the destination – does it really matter if we all go on the same path to get there?”

#### Towards a synthesis

If the “best practice” framing is accurate, then a synthesis of local and regional perspectives can be easily articulated: effective spread demands a balance between standardization and customization. However, the data suggested that this framing was misleading: whereas participants recounted success stories of the regional scale-up of *non-flow-related* best practices (notably the WHO’s Surgical Safety Checklist), flow-related best practices did not seem to exist; they either had not been identified, were disputed (quite understandably, given the weakness of the evidence base), or were so vaguely conceptualized that different sites’ versions could easily conflict with each other or with regional strategy. As a result, the Region sometimes found itself in the ironic position of squelching its most enthusiastic champions of change; their innovation was its aggravation. This was the actual substance of the conflict.

#### Thesis and antithesis clarified

Participants from hospitals and community areas described regional improvement efforts as slow and cumbersome, and decried the Region’s lack of support for site-led innovation. One recalled attempting to move ahead locally with an initiative that the Region was slowly developing: “I was told to stop. Everyone had to catch up...Everything has to be done regionally. So how’d that work out? …nothing changed, our numbers are awful. Worst in the country.” Another echoed, “I think the insistence on homogeneity stifles innovation...the system regularly stalls us.”

Participants from programs – especially non-acute programs – countered that site-led innovations might duplicate, misalign with or even obstruct regional strategies, and that chronic local variability might create inequity, as well as confusion, for patients and staff:

- “I think all too often we can run off and do a project – ‘that’s my project’ – without thinking, how does that little bit of implementation...link with the strategy of the region. I don’t think you can deal with patient flow unless we think strategically.”
- “…without a kind of consistent vision being overlaid over top, it can cause some disconnect. And really, I wouldn’t want to see somebody in [one community area] getting this much more service than somebody in [another], just because geographically...there’s not the same initiative in place.”

Of particular note were instances in which site and program/regional participants expressed contrasting perspectives on the same event. For example, participants disagreed about whether it created a problem for the larger system when one hospital unilaterally redesigned a particular process instead of awaiting the completion of a multi-year regional-level review:

- Site participant: “If you’re spending all your energy and saying, ‘Well your process is different than the regional process’ – Who cares, as long as the person is getting [served] in a timely fashion?”
- Regional participant: “Did the patient get [served]? Well – maybe; but...we’re a big system, and you have to have role clarity, and you know, what do we have resources hired for…You just can’t do things in isolation because you decide it’s better or quicker or safer…that’s an organizational risk.”

In another case, the launch of a site-led initiative provoked an acrimonious multi-year battle (still unresolved), although no one appeared fundamentally opposed to the initiative in principle.

#### Discussion

Clashes of the “Your Innovation Is My Aggravation” variety centred on the issue of who was authorized to lead change; they typically concerned the form of change rather than its substance. Conflicts not involving profound philosophical differences may be ameliorable through such remedies as role clarification and process enhancements. However, the very fact that relatively superficial disagreements frequently ballooned into rancorous major feuds raises the question of whether some deeper source of site–program strife was creating a polarized atmosphere; under such conditions, process solutions may be of limited value.

Comprehensive analysis of site–program conflicts (discussed below) revealed that the two groups did indeed hold inherently contradictory perspectives on the organization of care, and hence on the nature of flow solutions. This essential discordance, while not reflected in every site–program quarrel, may be key to understanding the pervasiveness and severity of conflict. It is at the core of the third paradox.

### Paradox #3: Your Order Is My Chaos

#### Thesis and antithesis

The fundamental axis of difference between sites and programs lies in the way that they define their patients: sites define patients in terms of *location in the system* (my patients are those at my site, not elsewhere), whereas programs define them in terms of *needs or characteristics* (my patients are those who need X, not something else). No participants explicitly articulated this disjunction; however, it lay just beneath the surface of many accounts of site–program conflict. The following two examples illuminate this.

##### Example 1: A conflict about site practices

In 2007, Hospital A undertook ED transformation with dramatic results; for years thereafter, this site maintained the lowest ED LOS for both admitted and non-admitted patients. As part of its efforts to maintain flow from the ED, it adopted the practice of assigning patients to beds irrespective of program; elective orthopaedic surgery beds, in particular, were frequently used as overflow to make room for new emergent admissions instead of for surgical patients. But when the Region consolidated orthopaedic surgery, Hospital A’s orthopaedic beds were converted from elective to emergent. While the consolidation succeeded in improving the timeliness of hip-fracture surgery at the regional level, it brought unintended consequences for Hospital A: At one fell swoop, the hospital gained responsibility for a large influx of emergency patients requiring admission, and lost its unofficial overflow beds. Inpatient wards were stretched to capacity – and for the first time in years, the ED became clogged with admitted patients. Both admitted and non-admitted LOS crept upward and did not recover.

What is instructive about this example is that, depending on whether the frame of reference is the hospital or the program, such bed-assignment practices can be viewed as either helping or hindering flow. From the site perspective, the use of surgery beds for non-surgical patients facilitated the efficient organization of care for patients occupying a given *location* (i.e., Hospital A): “If there’s a patient in our ER that requires a bed, they will get a bed... [Other sites] have silos within [the] building…we [have broken] down those silos, so that the beds aren’t owned by any specific discipline.” From the program perspective, Hospital A enjoyed this flexibility at the expense of other hospitals, impeding the efficient organization of care for patients with a given *need* (i.e., orthopaedic surgery): “...and they say, ‘our surgery beds are full of our medicine patients, so no, we don’t want to be using our surgery beds for surgery.’...That isn’t okay, that you don’t have space for your surgery patients and someone else is overheated.”

##### Example 2: A conflict about program “gates.”

Several participants observed that some programs seemed to function better than others; among these, Critical Care tended to receive approbation even from participants whose sympathies generally lay with sites. However, one site-based participant contended that Critical Care’s stringent regulations adversely impacted the care of patients who did not quite meet the program’s admission criteria:

- “So because they’re so busy protecting their beds...people come into Emergency, need critical care, get sicker because they’re not sick enough from a critical care perspective, sit in Emergency wasting those resources...and then you’re wondering why your flow is poor. Meanwhile...we have beds at our facility that those patients could go into...but we can’t gain access...and the person that’s losing out is the patient.... I think...charity begins at home, you gotta worry about who you have in front of you...[or] you’re not doing the best service for the patient that you’re trying to care for that’s in your site or your department or your unit.”

Conversely, a participant who espoused the “program” perspective maintained that such regulations were indispensable to ensure access for patients who *did* meet the admission criteria:

- “Before regionalization, community hospitals’ intensive care units were not functioning very well... it was a ridiculous waste of resources – everybody decided that they would keep an empty bed and dumped the next patient [from] the emergency room...to the teaching hospitals... And if you were a physician out in [a rural area] looking for a critical care bed in the city, you’d have to phone one guy after another and...they wouldn’t answer the phone or they’d tell you, ‘Sorry, we’re full, we don’t have any empty beds’ – and that was okay. And nobody felt responsible for these critically ill patients! Nobody.”

While both these participants appealed to values of efficiency and patient-centeredness, they offered different constructions of who the patients were: crucially, one defined patients by location (site/department/unit), the other by characteristics or needs (critical illness).

As this example illustrates, programs that define patients by characteristics/needs inevitably set up a “gate” that admits only patients possessing those characteristics/needs. Some programs’ criteria seem so self-evident that their gate escapes comment – no one would expect the Renal program to serve non-renal patients, or the Women’s program to serve men. In contrast, controversy exists about how rigorously, or by whom, the admission criteria of other programs – especially Medicine – should be applied (e.g., Should inpatient departments be compelled to admit “orphan” patients who meet no department’s criteria? Should Emergency physicians have admitting privileges?). Proponents of the site perspective, which defines patients by location, might question whether such criteria should exist at all:

- “Internal Medicine will always succeed. And the reason why it will succeed is because they’ve got the gate... I think Medicine will be very successful. Okay? But I think that the hospitals that the Medicine [program] is at will not be. ...I mean, I totally – I’d do the same... if I had the ability to put a gate at my [hospital] door, I would, I just don’t have that ability.”
- “The third [cause of poor patient flow] is some of the very rigid rules that some programs have in regards to consultation of patients.... A lot of rules, and nobody sees the patient as, it’s a patient that needs help.”

As noted earlier, the opposing view is that clear criteria are what make programs work well, often for a very sick or vulnerable group of patients, and that to weaken the gate would compromise such patients’ care.

#### Towards a synthesis

Leutz’s axiom “your integration is my fragmentation” expresses the idea that integration of some areas necessarily comes at the expense of others [25]. This study’s findings suggest an even broader principle, Your Order Is My Chaos: When there is overlap but not complete concordance between the way you and I define “our” patients, the definitions and rules that improve the organization of care for mine are likely to create obstacles for yours. This problem may potentially arise among different sites or different programs, but it *necessarily* arises between sites and programs because the two define their patients in fundamentally different ways: one by location, the other by needs. This disjunction manifests itself in perennial conflict about program gates.

##### Gated and ungated programs

Now that the paradox has been explicated in terms of a conflict between gated and ungated entities, another crucial dimension comes to light: the ungated entity can be a program. Family Medicine has a much weaker gate than does Medicine, and Emergency does not have one at all. As one participant put it: “Emerg can’t say no – your walk-in clinic can say no, your family physician can say no, your community agency can say, ‘No, sorry, you don’t meet our criteria’; Emerg can’t say no.” Not only does Emergency lack the ability to define its patient population, but this population has become larger and less differentiated over the past decades. Whereas the Casualty or Accident & Emergency departments of the past served a highly specific function, the modern ED is where patients with a plethora of primary- and specialty-care needs come to be “sorted.” As one senior executive quipped, “Emergency might as well be called Canada Post.”

Interview findings suggested that strongly gated programs were more regionalized; as well, they tended to be perceived as better-functioning. Although no participant made this connection explicitly, all programs that were praised as well-functioning had strong gates (i.e., served a clearly defined patient population), whereas those cited as poorly functioning had weak gates or none. It is easy to see why this might be: While it is possible to develop a path of care to efficiently meet the needs of a clearly defined population, it is very difficult to map, let alone improve, the process of care for one that is heterogeneous and amorphously defined. Whereas gated programs meet a specific need and can exclude patients not exhibiting that need, an ungated program receives considerable traffic from patients who are essentially *en route* elsewhere and need to be connected to the appropriate service. If these vital connections, which are outside the program’s control, cannot be made in a timely manner, the whole functioning of the ungated program breaks down.

##### Options for service design

If its lack of a gate renders the Emergency Program powerless to improve flow, what are the implications for system redesign? There are two options: Give it power or give it a gate. Option 1 (“Removing Gates”), based on defining patients by location, is to empower EDs to move patients through the hospital without regard for program gates. Option 2 (“Establishing Gates”), based on defining patients by needs, is to change the service model so as to ensure appropriate gates for every type of patient – and, in the process, to delimit the role of Emergency to the point that it effectively becomes a gated program. Option 1-type measures provide ways for ED physicians to admit or “send upstairs” patients who do not meet any program’s criteria (e.g., one-way consults, “uncapped” overcapacity protocols, hospitalist-staffed inpatient wards for unclaimed patients). Option 2-type measures involve directing specific types of patients to non-ED services, either before they present at Emergency (e.g., cross-sectoral care pathways that bypass the ED; expanded opportunities for scheduled urgent care) or immediately upon triage (e.g., diversion to ambulatory clinics, direct admission to inpatient programs).

There is local evidence that Option 1 can reduce ED congestion – albeit at a price, as in the example of Hospital A. On the other hand, it is difficult to judge the merits of Option 2 from the piecemeal initiatives the Region has undertaken. Certain initiatives have successfully diverted specific, small groups of patients from the ED, but with barely perceptible effects on overall volumes; meanwhile, community-based facilities introduced as ED alternatives have tended to attract new clients rather than avert ED utilization. Genuine implementation of Option 2 would involve radical change to the organization of care, the like of which has not been attempted in the Region or similar health systems. In the face of so overwhelming a prospect, it may seem safest to choose Option 1 – empowering sites to move patients as they see fit, and weakening program gates accordingly. But there is an inescapable flaw in this reasoning: namely, that it makes much less sense, *prima facie*, to define patients by location than by needs. Location is not an intrinsic property of patients; they merely present to whatever locations the system makes available to them. It is more logical – and patient-centered – to design services to fit patient needs than to expect patient needs to conform to available services.

#### Thesis and antithesis clarified

Options 1 and 2 each had their proponents: the former was preferred by site and Emergency stakeholders, the latter by other programs. However, the principle of basing service design on the needs of a clearly defined population was upheld by participants across the site–program spectrum: stakeholders representing sites as well as diverse programs related success stories about initiatives that did so:

- “There were different programs involved at the beginning [with] different views of what the solution would be, but once the focus was on the population, the solution was more successful, because it was created based on what was really needed.”

Furthermore, while the champions of Option 2 under no circumstances advocated removing gates, the strongest proponents of Option 1 *did* sometimes recommend establishing them. For example, when discussing how to improve the flow of non-admitted patients, Emergency stakeholders often endorsed rigorous enforcement of criteria and rules defined around patient needs (e.g., no use of Minor Treatment Area [MTA] spaces for non-MTA patients; no in-ED testing except to assess whether to admit; “powerful” admission and discharge criteria for short-stay units, etc.). Such recommendations implicitly acknowledge that when a service is extended beyond its intended population, its ability to serve that population is compromised. Only when recounting the difficulty of moving admitted or “can’t-go-home” patients out of the ED did such stakeholders define patients by location and object to the gates maintained by other programs. This suggested that their objections reflected, not philosophical opposition to population-based service design, but frustration at the lack of available services for some populations. For when patients are deemed not to “fit” the existing gates, it is hospitals – and EDs in particular – who are stuck with the conundrum of how to help them:

- “And [the admitting services] tell us, ‘Okay smarty-pants, sure, we’ll fill our beds with those people and there’ll be no room for really sick people, is that what you want?’ And we say in exasperation, ‘I don’t give a shit. They need to be cared for somewhere, they can’t go home, and my waiting room is full of patients at risk, so get them out of the emergency department!’”

The above analysis offers a new lens for interpreting participants’ comments about system (dis)organization and the need for fundamental system change. Those who described the system as in need of redesign often implied that gates had been established haphazardly, not as a systematic response to population needs. One remarked, “We have hundreds, thousands, if not millions of different people seeking services of various kinds…[at] maybe 2000 different doorways in our system. The result is chaos.” Another charged that program and service criteria had been designed on the basis of provider preferences, leaving so many gaps in the system’s ability to meet patient needs that it had created a “refugee situation – patients have to go to a refugee camp that’s called Emergency. And if they can’t be helped within a few hours, then they are admitted to the refugee camp called ‘the beds.’ Not necessarily because they need a bed; it’s because we haven’t been able to solve their problems.”

#### Discussion

Confronting the powerful image of “refugee camp medicine,” one’s immediate reaction may be to demand why some “country” (i.e., program) does not take in refugee patients. Closer examination, however, reveals the more fundamental question to be, Why are patients stateless in the first place? The answer is that these patients, for whatever reason, are unable to access services targeted to their specific needs.

If statelessness only befell a handful of patients, it might make sense to compel some program or other to accept them, or to create a “warehouse” for them, as per Option 1. But the pervasiveness of the refugee situation bespeaks a fundamental problem of system design. Where there is misalignment between service offerings and population needs, patients cannot reliably be directed to “the right place,” because the right place may not exist or may be filled with the wrong patients. Consequently, efforts to relieve congestion in one area often do little but move the congestion to another. Thus, the more strenuously programs and (especially) sites strive to create order in their own parts of the system, the more likely they are to create chaos in someone else’s. This is the deepest paradox of patient flow.

### General discussion

The three paradoxes reveal a system perpetually at odds with itself. Attempts to fix isolated components amounted to “Many Small Successes and One Big Failure.” However, a system approach was precluded by intractable conflict over who should lead change (“Your Innovation Is My Aggravation”) and, more fundamentally, whether such change should entail establishing gates or eliminating them (“Your Order Is My Chaos”). Unless it can resolve these core issues, the Region seems highly unlikely to achieve improved flow.

The findings illuminate why the Region was unable to mount a system response to the two key inhibitors of swift, even flow: [11] bottlenecks (Paradox 1) and variability (Paradoxes 2 and 3). Litvak and Long (2000) distinguished between artificial variability (arising from individuals’ preferences, e.g., surgeon-driven day-to-day variation in the elective surgery caseload), which should be eliminated, and natural variability (arising from differences in patients’ clinical profiles, patient arrival rates, and clinicians’ capabilities), which must be managed [17]. This study uncovered several examples of artificial variability, notably site-to-site process variations that complicated the effort to organize care regionally (“Your Innovation Is My Aggravation”). An even graver problem was that the fragmentation of authority across sites and programs fractured the Region’s ability to manage natural variability, as sites were predisposed to dismantle the infrastructure for doing so (i.e., gates; “Your Order is My Chaos”). In contrast to classic examples of artificial variability, [26] the source of divergence in managers’ preferences regarding gates was not individual but structural.

The analysis concluded that patient needs are the most reasonable basis for service design. This in no way implies that site personnel who, in the course of service *delivery*, define patients by location are being unreasonable. When a patient presents who does not quite fit any existing service, sites make the entirely reasonable judgement that s/he will be better off in a clinical unit than an off-service bed, an off-service bed than the ED, and the ED than the waiting room; the patient would likely agree. But the practice of assigning patients suboptimally is not consequence-free; it impairs the system’s ability to care for patients who expressly need the specific service. Thus, conflict is built into the system, as the imperative of sites to obtain care for their patients clashes with that of programs to provide care for theirs. This antinomy can only be resolved at the level of service design, where it is appropriate and necessary to choose which principle should govern the organization of care.

The principle of population-based service design (“Option 2”) is highly congruent with current North American thinking on delivery-system transformation; Porter and Lee’s proposals for advancing the “value agenda” – in particular, the creation of Integrated Practice Units organized around defined population segments or medical conditions – are a prominent example [27]. However, progress towards realizing Option 2 may be undermined by “Option 1”- type measures adopted in the hope of gaining some immediate relief for congested EDs and hospitals. In the world of flow initiatives, the path of least resistance leads away from the system we need to create.

The conclusion that the ED’s role must be redefined and reduced is bolstered by international evidence: Of 15 countries, the few not reporting severe ED crowding were those that did not expect the ED to be all things to all people, but had robust systems for managing patients elsewhere [3]. It should be noted, however, that there may be a variety of ways to operationalize population-based design, each with different implications for the model of emergency care. Christensen et al. (2009) offer one provocative possibility, based on a distinction between three types of enterprises: solution shops, which diagnose and solve unstructured problems; value-adding processes, which produce defined outputs through a predictable chain of steps; and facilitated networks, which offer a platform for user interaction and exchange [28]. Within healthcare, they note, a different type of care is best served by each of these models: diagnosis by solution shops, most treatments by value-added processes, and chronic disease management by facilitated networks. They argue that whereas today’s hospitals and clinics attempt to provide all three types of care within a single model, creating unnecessary complexity and confusion, each model should operate separately to function efficiently. This prescription might actually entail some *expansion* of the ED’s role to encompass more diagnostic activities (perhaps through collaboration between Emergency and hospital physicians [29]). However, expansion need not be incongruent with the analysis presented in this paper; what is critical is that each part of the system be designed to meet a distinct population need.

This study’s findings are also in line with the management literature, which has long associated matrix structures with organizational conflict [30]. Recent research has clarified that what produces conflict is not the presence of overlapping hierarchies *per se*, but of interdependent business units with discordant goals; strong superordinate goals may prevent matrix-related conflict from becoming destructive [31]. The Region’s leaders hoped to rally sites and programs behind the common cause of improving flow; what they appeared not to realize, however, was that a shared aspiration is not the same as a shared *direction*. As site and program solutions were often fundamentally incompatible, Senior Management’s assertion that both groups should be accountable for improving flow only fuelled conflict, with each group vying for the power to bring about the outcomes for which it was putatively responsible. Stronger accountability mechanisms, advocated by some participants, would seem of dubious benefit so long as different groups are held accountable for contradictory things. The Region’s problem of unclear accountabilities, then, reflected a deeper problem of unclear system goals: In the absence of clear goals, it will never be possible to define what individuals ought to be accountable *for*.

Although matrix management poses particular challenges, it does not follow that abandoning this structure is the answer. For the Region, abolishing the matrix would likely mean reversing any progress towards region-wide integration and returning to the arrangement of vesting power in sites, which might reinforce the counter-productive tendency to define patients by location instead of needs.

This study has certain limitations. The first is its reliance on a single analyst. While it is optimal to use multiple coders, it is important to note that extensive participant validation ensured the incorporation of multiple perspectives. Following circulation of the draft report, fully one third of participants took the opportunity to provide feedback, and several engaged me in substantive discussion over e-mail or in person. These conversations, and the revisions they occasioned, continued until participants appeared satisfied that the report represented the facts and competing viewpoints accurately and fairly. This process did much to enhance the trustworthiness of the analysis. A second limitation is that my quasi-insider status as an embedded researcher may have biased the interpretations even while enriching them – although it clearly did not inhibit me from delivering “bad news.” Thirdly, although the sample was diverse, representing different organizational levels and clinical/non-clinical backgrounds, it was restricted to management. This focus was deliberate, as managers are best placed to observe macro-system dynamics; in fact, the greatest proportion of data on misalignments among system parts came from the highest levels of management (program, site and regional leadership). Nonetheless, frontline staff and patients might have offered valuable illumination of other dimensions of the flow problem. The greatest limitation was that the study concerned a single organization, whose system design and performance remained basically stable over the study period. We know that this is not the only Canadian region to have pursued multiple flow-improvement interventions with little success, [32] but we do not know whether other systems have encountered the same or different paradoxes, or how they might have addressed them. Future research should include an intervention study of a major system-redesign effort in the Region and/or a comparative case study of jurisdictions with varying levels of performance. Research should also examine the extent to which population-based service design may be a factor in the success of high-performing health systems.

## Conclusion

The attempt to improve flow by sponsoring multifarious projects in different parts of the system is often mistakenly equated with a “system approach.” However, when goal conflict is built into the system, such piecemeal efforts are inevitably defeated by the Three Paradoxes of Patient Flow. A true system approach would entail defining each patient population by needs, analyzing its entire trajectory of care, and establishing a consistent process that better meets those needs, whether by revising an existing service model or creating a new one. Although fundamental system change is inevitably difficult, resource-intensive, and disruptive, it offers our best hope of achieving real improvement.
